# Computational Approaches for Predicting Biomedical Research Collaborations

**DOI:** 10.1371/journal.pone.0111795

**Published:** 2014-11-06

**Authors:** Qing Zhang, Hong Yu

**Affiliations:** 1 Department of Quantitative Health Sciences, University of Massachusetts Medical School, Worcester, Massachusetts, United States of America; 2 VA Central Massachusetts, Leeds, Massachusetts, United States of America; University of Illinois-Chicago, United States of America

## Abstract

Biomedical research is increasingly collaborative, and successful collaborations often produce high impact work. Computational approaches can be developed for automatically predicting biomedical research collaborations. Previous works of collaboration prediction mainly explored the topological structures of research collaboration networks, leaving out rich semantic information from the publications themselves. In this paper, we propose supervised machine learning approaches to predict research collaborations in the biomedical field. We explored both the semantic features extracted from author research interest profile and the author network topological features. We found that the most informative semantic features for author collaborations are related to research interest, including similarity of out-citing citations, similarity of abstracts. Of the four supervised machine learning models (naïve Bayes, naïve Bayes multinomial, SVMs, and logistic regression), the best performing model is logistic regression with an ROC ranging from 0.766 to 0.980 on different datasets. To our knowledge we are the first to study in depth how research interest and productivities can be used for collaboration prediction. Our approach is computationally efficient, scalable and yet simple to implement. The datasets of this study are available at https://github.com/qingzhanggithub/medline-collaboration-datasets.

## Introduction

Millions of researchers contribute to biomedical research, collectively publishing tens of millions of research papers. These research papers interlink researchers into a complex co-authorship network. Biomedical research is a fast-growing interdisciplinary field that frequently requires high degree of collaboration. It has been found that the average number of collaborators in the biomedical field is twice that in physics and more than four times that in mathematics [1]. Such collaborations span basic, translational, and clinical research. Successful collaborations often yielded high impact work [2]–[5] such as the Gene Ontology [6].

The importance of scientific collaboration has motivated the development of researcher profile platforms, most of which focus on facilitating institutional collaborations. Such platforms, including the Harvard Catalyst Profile [7], SciVal Experts [8], and ProQuest Pivot [9], integrate research and collaboration information—including publication history, co-authorship connections, research topics, and funding information—making it easier to find potential collaborators. In addition, semantic Web resources, including VIVO [10], have been developed to provide a general scheme to describe researcher profiles so that the profiles can be embedded in particular applications. Online communities, including BiomedExperts [11], allow users to upload their personal profiles and help them make new connections. Few systems, however, have the functionality of recommending collaborators automatically. Such services, on the other hand, may be important for researchers, especially junior researchers, whose work depends upon successful collaborations. Automatically recommending collaborators may offer an attractive alternative to traditional ways of finding a collaborator, such as socializing at a scientific conference or being introduced by a mutual colleague.

**Figure 1:**
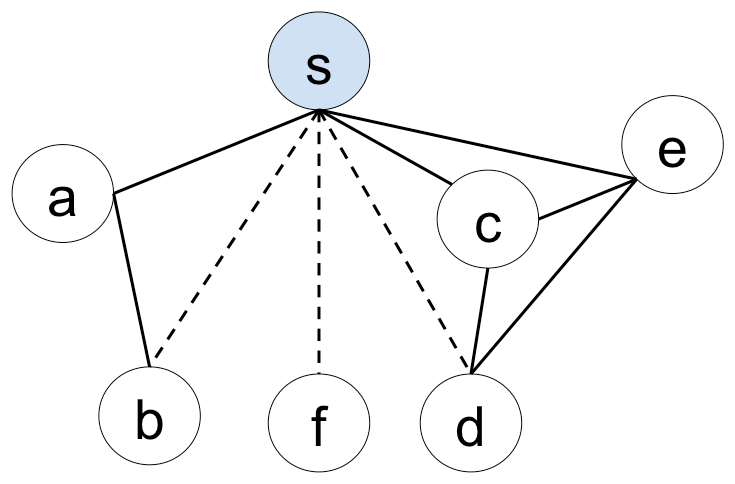
An illustration of automatic research collaboration recommendation. The graph shows a co-authorship network in which the nodes are authors and the links represent co-authorship. The solid lines represent existing co-authorships. Our study is to build a computational model to predict whether author *s* will collaborate with authors *b*, *f*, and *d* based on their existing research and collaborations.

We formulate research collaboration prediction as a link prediction problem in the context of a co-authorship network. Since joint publication is one of the most effective representations of collaboration, a co-authorship indicates a collaborative relation. Our goal is illustrated in Figure 1, where author *s* has collaborated with authors *a*, *c*, and *e* and we would like to know the probability that *s* will collaborate with *b*, *f*, and *d*.

Link prediction has been studied in social networks. Liben-Nowell and Kleinberg [12] used various topological features for link prediction. For example, two researchers are more likely to collaborate with each other when they have common collaborators. Al Hasan, et al. [13] compared different machine learning models and learning features for predicting author collaboration. They found that support vector machines (SVMs) performed the best and shortest distance (i.e., the minimum number of edges that separate two authors) is a top topological feature. In addition, they explored node attributes (e.g., author's productivity and the research similarity between two authors) as additional features and concluded that they are top features for the prediction. Backstrom and Leskovec [14] explored a supervised random walk model and found it outperformed decision tree and logistic regression models in predicting new friends in Facebook. The aforementioned work provides important understanding of link predictions problem formulation. However most of the work mainly explored network topological structures. On the other hand, the rich semantic information (research work and collaboration activities), well documented by their publications, have been explored little.

In this paper we report the development and evaluation of Automatic Research Collaboration Recommendation (ARCR) system to predict new author collaborations. ARCR was built on supervised machine learning models. Our contributions are: We explored rich learning features, which were derived from the semantic content of an author's research profile. Our supervised machine learning models and learning features are computationally efficient, making them applicable to the big data challenge of scientific collaboration recommendation. In addition, we evaluated our approaches on various datasets reflecting data sparseness, a common problem in the real world. Finally, we provided in-depth analysis of important features including research interest and mutual collaborators that contribute to biomedical collaborations.

## Background

Author collaboration prediction can be considered as a case of modeling evolving networks. Significant amount of theoretical work is based on network structures and their evolution. Early work modeled the network as random graph, where the establishment of a connection follows Poisson distribution [15]. Later the scale-free model was proposed, in which the probability of a new node connecting with a given node is proportional to the degree of the node [16]. This phenomenon, which is also called preferential attachment, has been observed in many evolving networks, including social networks [1], [17], World Wide Web [18], and the protein-protein interaction network [19]. Additional findings, including “first-mover-advantage” [20] and “the-fit-get-richer” [21], have enriched further the scale-free model.

“Small world” is another important characteristic in various networks [22], [23]. A social network, including a co-authorship network, consists of both structured (close neighbors) and random contacts and one can navigate from one node to another with very few steps. Newman [17] found that only five to six steps are needed to navigate from one randomly chosen scientist to another in a community. In addition, social networks appear assortative, meaning that nodes tend to connect to other nodes with similar characteristics (e.g., the degree [24]).

In computer science, author collaboration prediction is often formulated as a link prediction problem. Early work focuses on topology-based prediction that utilizes network structure, including the connectivity and similarity of neighbor nodes. As stated earlier in this paper, Liben-Nowell and Kleinberg [12] comprehensively evaluated a collection of topological predictors, including number of common co-author and random walk, for the link prediction in co-authorship network of physics field. The work is one of the foundations of many later studies[13], [14], including ours.

Much work in link prediction explored supervised machine learning models and different learning features. As stated earlier, Al Hasan et al. [13] explored naïve Bayes and SVMs. They explored topological features (e.g., the number of common co-authors) and simple semantic features (e.g., the overlap of the keywords of two author's publication profiles). Sun et al [25] studied topological features in the heterogeneous networks consisting both co-authorship and citation relations to predict co-authorship in the DBLP data sets. Backstrom and Leskovec [14] applied supervised machine learning to predict the strength of a connection. The predicted weight is subsequently used to guide the random walk. The stationary probability of landing on a particular node is considered as the chance of a connection from the starting node. Wang et al [26] modeled the local topological structure by Markov Random Field to infer the co-occurrence probability of two nodes, and subsequently integrated with other topological and semantic features for link prediction.

Co-authorship networks have been widely studied. For example, Newman [17] compared the co-authorship network in biomedicine with that in physics and observed differences. He made several observations. In the biomedical domain, it is less common that two researchers collaborate when they have a mutual collaborator than in physics. The networks are scale free: the network structure is dominated by many “little” people with few collaborators, instead of a few people with many collaborators. He also observed that two researchers are more likely to collaborate if they have had a strong history of collaborations, either between themselves or with others) [27].

Several studies showed that co-authorship networks in the biomedical domain exhibit different characteristics than network in other domains. Newman [1] showed that biomedical research has the highest degree of collaboration, in comparison with the physics and mathematics domains. Huang, et al. [28] observed that the collaboration pattern and its evolution in the computer science domain are more similar to the mathematics domain than to biology. Ding [29] found that, in the information retrieval field, productive authors tend to collaborate with and cite researchers who have the same research interests.

Factors that lead to successful collaborations have also been studied, including various social and environmental factors: leadership, geographical proximity, and the personalities of the team members. For example, one study concluded that a leader in a research field typically plays an important “broker” role to bridge people from different disciplines [5]. Physical proximity between first and last author was found to be positively related to the impact of collaboration, measured by the citation received [30]. They concluded that close geographical distance is important for the outcome of the collaboration. International collaborations, however, are found to be a positive factor for the impact of a work. As shown in [31], the average number of citations increases with the number of affiliated countries. Certain characteristics of team members, such as openness and flexibility, also contribute to the success of the collaboration [5], [32].

Our work is closely related to the work of Al Hasan, et al. [13]. However, unlike their approach which mainly explored topological features, we explored rich semantic features derived from the author's research profile, including publication history similarity, citation similarity, and common co-authors, and we show that these semantic features significantly improve the research collaboration predictions.

## Materials and Methods

We formulate research collaboration prediction as a classification task and therefore explore supervised learning approaches. In the following we first describe the supervised machine learning models we used and then the feature set.

### Supervised Machine Learning Models

We explored four supervised machine learning models: naïve Bayes, naïve Bayes multinomial, Support Vector Machines (SVMs), and logistic regression, which are all commonly used for classification tasks. A naïve Bayes classifier is a probabilistic classifier based on Bayes' theorem with the naïve assumption that the features are independent from each other, given the instance label [33]. The naïve Bayes multinomial model assumes the conditional probability of the feature, given a class, follows a multinomial distribution [34]. SVMs are based on the concept of maximum margin decision planes that define generalizable decision boundaries for classification and regression. An SVM constructs a hyperplane to maximize the margin between the data points and the hyperplane, often after mapping the data points to a higher-dimensional space in which they are linearly separable or close to it [35]. We explore an SVM model with the widely-used linear kernel for its efficiency. Logistic regression estimates discrete or continuous value parameters to predict discrete category values. The probabilities that describe the possible class of a single instance are trained as a function of explanatory variables, using a logistic function[33]. These four classifiers are not only the well-studied models in a variety of classification tasks [36], but also widely available in open source software communities. In addition we used K-nearest neighbor model (KNN) as it particularly learns non-linear decision boundaries and is easy to interpret [36], [37]. We use data mining software Weka [38] to build and evaluate naïve Bayes, naïve Bayes multinomial and logistic regression models, LIBSVM [39] for SVM, and the python machine learning package Scikit [40] for KNN.

### Features

There are many reasons why two researchers collaborate, e.g., geographically close proximity (e.g., within or outside institutes) [30], proximity in the network (e.g., two researchers who have colleagues in common are more likely to collaborate) [12], and proximity in research (two researchers with the same goal in research may collaborate). Topological proximity has long been studied and considered as a factor of establishing connection in social networks. Semantic features, on the other hand, integrate specific domain knowledge of the nodes and have not yet been fully explored, which are the major contributions of this paper. In the following we will first describe the topological features, and then the semantic features.

#### Co-authorship Network Connectivity

Newman [1] observed that two scientists with a common collaborator are more likely to co-author a paper than two scientists who have no common collaborator. We therefore explored this feature called *numCommonCoauthor* of authors *x* and *y*, which is defined as
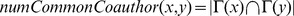
where 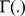 is the set of coauthors, and the feature value is number of co-authors two researchers have in common.

*coAuthorJaccard* and *Adamic* are two extensions of the common coauthor feature, both of which have been studied by Liben-Nowell et al [12]. *coauthorJaccard* is the number of co-authors two researchers have in common normalized by the total number of their unique co-authors. *Adamic* was first introduced by Adamic et al in [41] to measure the similarity of two web pages. The idea is that two web pages are more similar if they have common web pages that link both. Web pages that are exclusive to the two web pages are weighted more than those that also link to other web pages. *Adamic* was explored for link prediction [12], [13], although its contribution to link prediction remain inconsistent among different studies. While Liben-Nowell and Kleinberg [12] found that *Adamic* was one of the most valuable features, Hasan et al [13] did not report any performance improvement. Here we adopted *Adamic* to measure the similarity of two researchers by their common neighbors. For authors x and y, 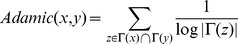
where z is the common neighbor (co-author) of *x* and *y*. The larger the value, the more similar *x* and *y* are. The higher number of the common co-authors is, the higher the *Adamic* value is. Each common co-author is also weighted by their exclusiveness to authors *x* and *y*. The less inclusive a common co-author is, the higher its *Adamic* value. Assuming that researcher z is the only common neighbor of *x* and *y* and that z has no other connections other than *x* and *y* (or *x* and *y* are the only co-authors of z), the *Adamic* value of *x* and *y* is 1/log(2). On other hand if z has 3 connections in addition to *x* and *y*, the value becomes 1/log(5). Therefore *x* and *y* are more similar in the former case.

A feature commonly used for describing the small-world characteristics in a network is clustering coefficient [27], which we designated the feature as *sumClusteringCoef*. It is the sum of each researcher's clustering coefficient, a measure of the probability that a researcher's collaborators have collaborations among themselves. The higher the clustering coefficient the closer the nodes in the network are connected.

We also included the feature *sumCoauthor*, which is the sum of each researcher's average number of unique co-authors per year. *SumCoauthor* represents how active a researcher is in collaboration with others, which is defined as
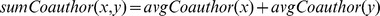
where *avgCoauthor(.)* is the average number of unique co-author per year.

#### Research Profile Similarity

It was reported that the keyword overlap from two author's publication history was more effective than topological features [13]. We therefore explored research profile similarity as additional features. To do so, we first built a research profile for every author. Specifically the research profile of an author comprises of three components of all his/her publications: abstracts, the assigned Medical Subject Headings (MeSH) terms, and the citations. We speculate that these components represent the author's research interests: abstract is the summary of an article by the author(s); MeSH terms represent main topics of the article; and out-citing citations (other articles cited by the article) show the relevant background information of the article while in-citing citations (other articles that cite the article in question) represent the recognition of the work by peers.

We used the classical vector space model (TF*IDF weighted) to build the research profile. Assuming that the publication collection of author *s* by a certain year is *D*, the TF-IDF for term *t* in *D* is calculated by 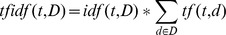
where *idf(t, D)* is the inverse document frequency of term *t* which is calculated from the entire MEDLINE database and *tf(t,d)* is the term frequency of term *t* in collection *D*. Using the aforementioned formula, we built three vector space models to represent abstracts, in-citing and out-citing citations, respectively. We did not compute the MeSH TF-IDF vector due to our preliminary study from which we found that the TF-IDF representation for MeSH terms did not improve the performance. Instead, we included all unique MeSH terms in the collection to represent an author's MeSH profile.

We then derive learning features from two authors' research profiles. Specifically we define features *simText*, *simOutcite*, and *simIncite* as the cosine similarity of two researchers' abstract profiles, out-citing citation profiles, and in-citing citation profiles, respectively. Concretely,
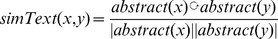
where *abstract(.)* is the TF-IDF term vector of the author's publication history. Similarly
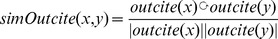
and
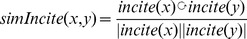
where *outcite(.)* is the TF-IDF term vector of the author's out-citing citations from the publication history, while *incite(.)* is the TF-IDF term vector of the author's in-citing citations of the publication history. We also define *simMeSH* as the Jaccard coefficient of the two researchers' MeSH profiles. Concretely,
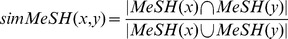



Where *MeSH(.)* is the MeSH terms of author's publication history.

#### Collective Productivity

The total number of publications of an author was explored in [13] and was shown effectiveness for predicting author collaborations. Here we use the average number of publications per year to measure productivity and *sumPub* as the sum of two researchers' average publications. Formally it is defined as
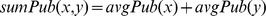
where *avgPub(.)* is average publication per year. In addition, similar to age effect [42] we attempted to increase the weight of recent year productivity and defined an author's *recency* as the sum of the inversed publication time distances to the present. This metrics will weigh-in most recent activity. The *recency* of author *x* is defined as
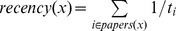
where *papers(x)* is the publications of the author x, and 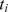
is the distance between publication year of paper *i* and the *present* year. The *sumRecency* is thus the sum of two researcher's *recency* scores.

#### Seniority

The relations between two researchers include junior–senior relations (e.g., student and advisor) and collegial relations, which may be important for research collaborations. We define an author's *seniority* as the average number of times that the author has been a senior author, which is approximated by the corresponding author in this study. The *seniority* of author *x* is defined as 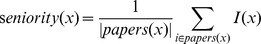
where *I(x)* is the indicator function; it equals one if x is the corresponding author of the particular publication, and is zero otherwise. For example, assume an author had 5 publications and was the corresponding author on 2 of them, thus the author's seniority is 2/5. The feature *diffSeniority* is thus defined as the seniority difference between two researchers. Table 1 shows the formal definitions of features we explored.

**Table 1:** Feature definition.

Category	Feature	Definition
Connectivity	*numCommonCoauthor*	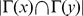 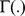 is the set of the researcher's co-authors (neighbors in the network)
	*coauthorJaccard*	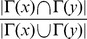
	*Adamic*	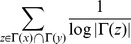 The idea is that two nodes are more similar if they share a lot of neighbors that mainly connect to these two nodes.
	*sumClusteringCoef*	Sum of both researchers' clustering coefficients
	*sumCoauthor*	avgCoauthor(x) + avgCoauthor(y) avgCoauthor(.) is the researcher's average number of unique co-authors per year.
Research profile similarity	*simText*	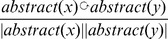 Cosine similarity of two researcher's publication history, measured by abstract TF-IDF term vectors
	*simOutcite*	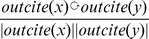 Cosine similarity of two researchers' out-citing citations' TFIDF term vectors
	*simIncite*	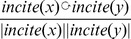 Cosine similarity of two researchers' in-citing citations' TF-IDF term vectors
	*simMeSH*	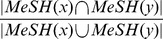 *MeSH*(.) is the MeSH term set of the researcher's publication history
Collective productivity	*sumPub*	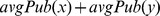 *avgPub(.)* is the researcher's average number of publications per year
	*sumRecency*	*recency(x)+recency(y)* and 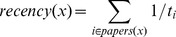 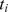 is the time difference between the publication date of paper *i* to the present, and *Paper* is the publications of author *x.*
Seniority	*diffSeniority*	*seniority(x)-seniority(y)* seniority(.) is the average number of times a researchers has been a senior author

### Baselines

The first baseline model, called *PreferentialAttachment*, is based on the Barabasi-Albert scale-free model[16]. As described in the background section, preferential attachment is a well-studied network growth pattern. The more existing links a node has, the higher the chance a new node will link to it. We implemented this baseline based on Liben-Nowell and Kleinberg's description [12]. Specifically 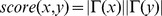, where 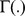 represents the set of neighboring nodes. For each pair of nodes *x* and *y* in a testing set, we computed the corresponding *score(x,y)*. The higher the score, the larger the chance that the two nodes *x* and *y* will connect (or collaborate).

We also used *JaccardBaseline*, which describes the importance of the common co-author in the author pair, as the third baseline model as it has demonstrated strong performance in previous research [18]. Its definition is the same as for the feature *coauthorJaccard*.

### Data

We used the citation/co-authorship network database CiteGraph [43] as the data source. The database comprises of 1.6 million full-text articles, a joint set of the Elsevier database (1899–2011) and the MEDLINE database. Each article entry includes the title, author(s), abstract, full text, year of publication, and the MeSH terms, as well as the in-cites and out-cites. We disambiguated author names and built a co-authorship network.

**Figure 2:**
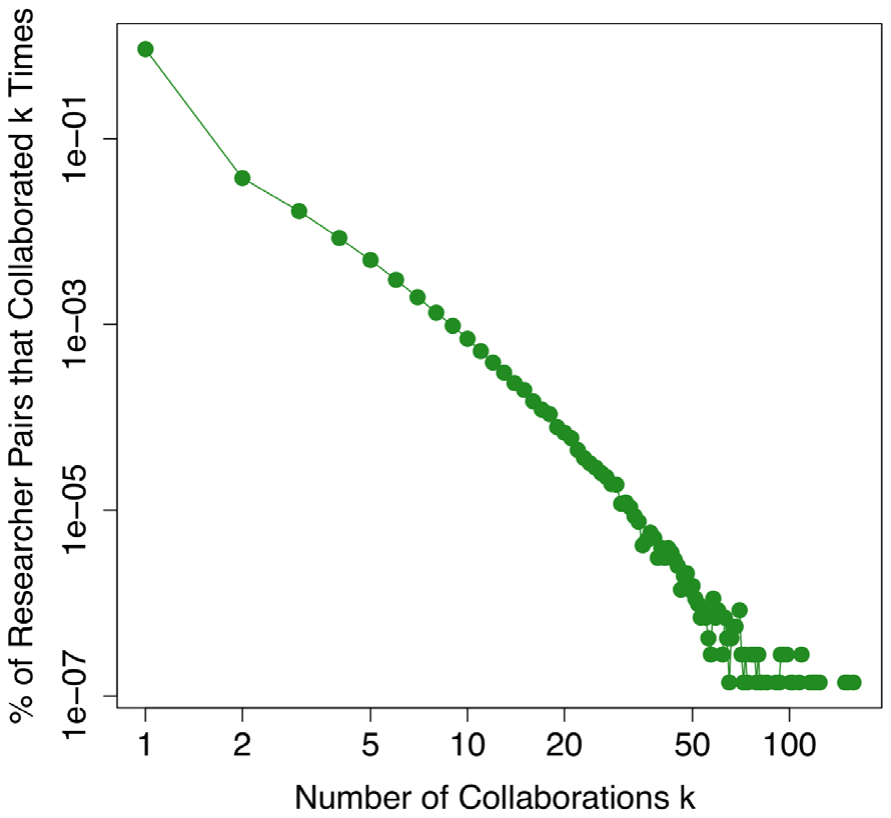
Collaboration frequency distribution for the CiteGraph dataset. It is a power law distribution log (*y*) = −3.59* log (*x*)+0.885, where *y* refers to the percentage of researcher pairs that collaborate *x* times, with x referring to the number of collaborations.

Figure 2 shows the collaboration frequency distribution in the CiteGraph dataset. As shown in the figure, an 80.5% majority of researcher pairs collaborate only once, while less than 20% collaborate two or more times. The highest number of collaborations for the same researcher pair is 159, spanning 12 years. The percentage, *y*, of researcher pairs that collaborate *x* times follows the power law distribution log *y* = −3.59 * log *x*+0.885, where *x* refers to the number of collaborations, with statistical significance (p<0.05, t-test) for the linear regression.

### Training Dataset

We used CiteGraph for both the training and testing data. We selected equal numbers of positive and negative instances for training and testing as such makes results more comparable to previous work [13]. The positive training instances are author pairs whose first collaborations took place in 2007 or 2008. The negative training instances are author pairs who did not collaborate before 2009. We randomly selected 10,000 positive and 10,000 negative author pairs and extracted each pair's features, and the sampling method is similar with the static graph sampling algorithm proposed in [44]. Since article information, including the abstract, was not available for some authors, we filtered out these pairs, resulting total of 5361 positive instances and 5361 negative instances. The combined group of 10,722 author pairs was used as the training set.

### Testing Datasets

We created two sets of testing data. The first set of data, *RandomPairCategory*, was created from a random selection of publications from 2009 and 2010 using the same sampling approach as training set. The positive instances were those in which the author pair first collaborated in 2009 or 2010, while the negative instances were author pairs who never collaborated before 2011. We randomly identified a total of 10,000 positive and 10,000 negative author pairs. Of these, we found that 4726 positive and 4726 negative author pairs had complete features. These 9,452 author pairs were used as the testing set. Note that the selection method of *RandomPairCategory* was utilized for the training data; therefore, the two datasets represent the same distribution.

The second testing dataset, *IndividualAuthorCategory*, was selected based on the collaboration network topology. We randomly selected four authors (target authors) with multiple publications (we set a minimum of 10) in 2009 and 2010. For each author, we built a sub-graph comprising three hops of a breadth-first traversal of the collaboration network established prior to 2011. We thus not only built a sub-graph, but also created the testing set with authors who are close topologically. The positive instances are collaborations established by authors (in the sub-graph) who collaborated with the target author during 2009 and 2010 and the negative instances are those (in the sub-graph) who did not collaborate with the target author before the end of 2010. The statistics of each sub-graph are shown in Table 2. When constructing the testing set for each author, we used all the positive instances and randomly sampled 200 negative instances for each author.

**Table 2:** *IndividualAuthorCategory* testing sets.

Author	Number of Publications	Sub-Graph Size	Positives	Negatives Sampled
Jeroen Bax	28	69,487	31	200
Mathew Farrer	10	66,876	13	200
Filippo Marte	59	418	11	200
Christodoulos Stefanadis	30	33,869	16	200

The *IndividualAuthorCategory* evaluation dataset complements the *RandomPairCategory* dataset because the former consists of author pairs who tend to be more similar in research while the latter represents a broader selection of potential collaborators.

We calculate precision (TP/(TP + FP)), recall (TP/(TP + FN)), the receiver operating characteristic—or ROC, the area under the curve of the true positive rate (TPR) over the false positive rate (FPR)—sensitivity (the same as recall), specificity (TN/(FP+TN)), and accuracy ((TP+TN)/ALL), where TP, FP, TN, FN and ALL stand for number of true positives, false positives, true negatives, false negatives, and number of total instances respectively. F1 score is defined as the harmonic mean of recall and precision, specifically 2*recall*precision/(recall+precision). In addition we use log loss[33] to measure the prediction cost of logistic regression model. It is defined as 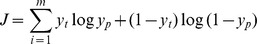
where 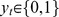 is class label, and 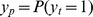 is the predicted probability of being positive.

### Feature and Research Profile Analysis

We analyzed the importance of features using information gain and feature value distributions of true positive (TP), false positive (FP), true negative (TN), and false negative (FN) predictions. We also studied how features' contributions evolve over time, as the authors presumably become more senior.

## Results

### 10-fold Cross Validation on the Training Set

Table 3 shows the 10-fold cross-validation results on the training dataset. The logistic regression and SVM demonstrated the best performance, with a 0.878 ROC and 0.797 F1 for logistic regression and 0.878 ROC and 0.780 F1 for SVM. The naïve Bayes model performs the second best, with an ROC of 0.838. The naïve Bayes multinomial performed the worst among the models. Logistic regression as well as SVM outperformed the naïve Bayes and naïve Bayes multinomial models with statistical significance (p<0.05, t-test).

**Table 3:** 10-fold cross-validation on the training set.

Model	ROC	Precision	Recall	F1	Accuracy
Naïve Bayes	0.838	0.798	0.708	0.684	0.708
Naïve Bayes Multinomial	0.659	0.795	0.655	0.609	0.655
Logistic Regression	**0.878**	**0.803**	**0.797**	**0.796**	**0.797**
SVM	**0.878**	**0.855**	**0.718**	**0.780**	**0.798**
KNN (N = 51)	**0.858**	**0.868**	**0.636**	**0.734**	**0.769**

### Testing Set 1

Table 4 shows the results of models that were trained on the entire training dataset and then tested on the *RandomPairCategory* testing set, which was created by randomly selecting author pairs published during 2009 and 2010. Consistent with the cross-validation results, the logistic regression and SVM outperformed the other models, yielding an ROC of 0.871 and an F1 of 0.789 for logistic regression and 0.871 ROC and 0.769 F1 for SVM. The topology baseline models *PreferentialAttachment* and *JaccardBaseline* yielded ROC values of 0.4583 and 0.278, respectively. All the supervised machine-learning models outperformed the baseline systems. Logistic regression outperformed the naïve Bayes and naïve Bayes multinomial models with statistical significance (p<0.05, t-test).

**Table 4:** *RandomPairCategory* evaluation results.

Model	ROC	Precision	Recall	F1	Accuracy
Naïve Bayes	0.819	0.786	0.694	0.667	0.694
Naïve Bayes Multinomial	0.626	0.790	0.644	0.592	0.644
Logistic Regression	**0.871**	**0.794**	**0.789**	**0.788**	**0.789**
SVM	**0.871**	**0.842**	**0.708**	**0.769**	**0.787**
KNN (n = 51)	0.850	0.854	0.632	0.726	0.762
*PreferentialAttachment*	0.584	0.574	0.567	0.556	0.567
*JaccardBaseline*	0.639	0.789	0.639	0.585	0.639

### Testing Set 2

We evaluated the top-performing supervised machine-learning model, logistic regression, on the *IndividualAuthorCategory* testing set, and the results are shown in Table 5. Our model yielded ROC ranging from 0.766 to 0.980, while the best ROC for the baseline models was 0.634 for the prediction for author Jeroen Bax for the *PreferentialAttachment* model; the *JaccardBaseline* model performed best for predicting collaborators of Mathew Farrer, with an ROC of 0.917. The performance differences between ARCR and the baselines are both statistically significant (p<0.05, t-test).

**Table 5:** *IndividualAuthorCategory* evaluation results.

Author	ARCR ROC	Pref. Attach.* ROC	JaccardBaseline ROC
Jeroen Bax	0.917	0.634	0.620
Mathew Farrer	0.980	0.537	0.917
Filippo Marte	0.800	0.302	0.455
Christodoulos Stefanadis	0.766	0.548	0.313
Macro Average	0.866′	0.505	0.576

*Pref. Attach stands for *PreferentialAttachment*.

### Inter- vs. Intra-discipline Collaboration

**Figure 3:**
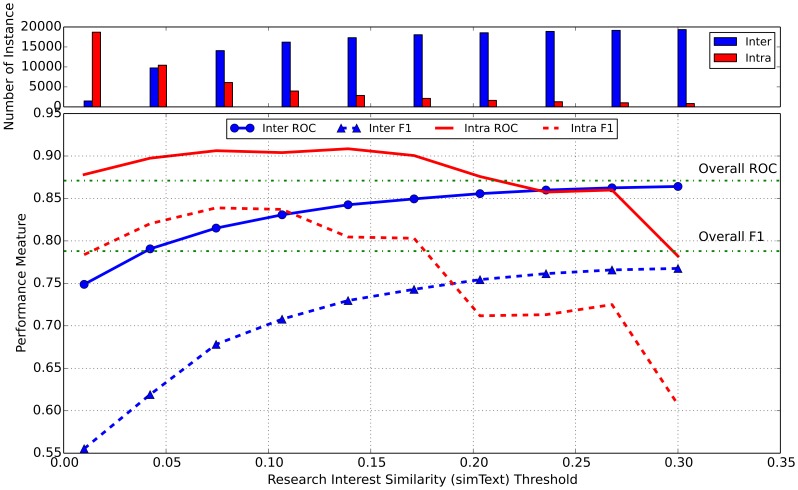
Intra and inter-disciplinary collaboration prediction performance by ROC and F1 measurement. Training set and *RandomPairCateory* test set were divided by the threshold into inter- (<threshold) and intra-disciplinary (>threshold) training/test sets. For each threshold, we trained inter- and intra-disciplinary models and tested them on the corresponding inter-/intra-disciplinary testing sets. The histogram on the top is the number of instances (training+testing) of inter- and intra-disciplinary subset according to the threshold cutoff. The ROC and F1 of overall data are also denoted as the two dotted horizontal lines.

We further examined inter- and intra-disciplinary collaboration predictions separately. Although *simMeSH* can be used as the discipline measure, we assume that the abstract has more detailed information than keywords. We therefore split the training data using different values of *simText* as our threshold in order to approximate inter-discipline and intra-discipline collaboration. The training set and the *RandomPairCateory* testing set were divided into inter-/intra-disciplinary training/testing sets using the threshold. We varied the threshold from *simText* values of 0.01 to 0.30 with 15 evenly distributed data points. For example, when the *simText* threshold was set to 0.01, author-pair instances with *simText* values less than 0.01 were categorized as inter-disciplinary whereas the author-pair instances with *simText* values greater than 0.01 were categorized as intra-disciplinary. For each threshold value, we trained inter-and intra-disciplinary learning models using their respective training sets and then tested them on the corresponding inter-/intra-disciplinary testing sets. Since there was very little data when *simText* is larger than 0.3, we did not explore larger thresholds. Overall the inter-disciplinary collaboration resulted in ROC and F1 ranging from 0.75–0.86 and 0.66–0.77 respectively across different thresholds. The intra-disciplinary prediction achieved ROC 0.78–0.87 and F1 0.61–0.77. As shown in Figure 3, when *simText* <0.19, the intra-disciplinary model yielded a better performance according to F1. When *simText* was over 0.19, the inter-disciplinary model outperformed the intra-disciplinary model. The inter-disciplinary collaboration in general under-performed the intra-disciplinary one, suggesting it is more difficult to predict former and there might be other potentially important factors that influence the inter-disciplinary collaboration. Although there is no absolute *simText* value to divide the inter-/intra-diciplinary collaboration, as a reference our preliminary study shows that *simText* 0.1 represent distant topic (research interest) pair such as *diabetes* and *gene regulation*, while more related topic pair *brain* and *Alzheimer* has *simText* 0.3.

### Feature Ranking

To identify the features' contributions, we ranked them using information gain [45]. As shown in Table 6, the research interest features *simOutcite* and *simText* are the top-ranked features, both with information gain greater than 0.2. The features *coauthorJaccard*, *Adamic*, and *numCommonCoauthor* are the next top ranked, based on the common co-author count. The next features are *simMeSH* and *simIncite*, which also represent research interest. In contrast, the contributions of *sumClusteringCoef* and *sumPub* are considerably smaller and *diffSeniority* shows no contribution.

**Table 6:** Training set feature ranking, by information gain.

Rank	Feature	Information Gain
1	*simOutcite*	0.265
2	*simText*	0.202
3	*coauthorJaccard*	0.173
4	*Adamic*	0.173
5	*numCommonCoauthor*	0.173
6	*simMeSH*	0.145
7	*simIncite*	0.101
8	*sumCoauthor*	0.055
9	*sumRecency*	0.024
10	*sumPub*	0.022
11	*sumClusteringCoef*	0.002
12	*diffSeniority*	0

In order to analyze error patterns across different datasets, we calculated sensitivity and specificity for the logistic regression model evaluation on all the testing sets. As shown in Table 7, the *RandomPairCategory* testing set has lower sensitivity than the *IndividualAuthorCategory* testing set, while the former has higher specificity than the latter.

**Table 7:** Sensitivity and specificity for all testing sets by logistic regression model.

Testing Set		Sensitivity	Specificity
RandomPairCategory		0.718	0.859
IndividualAuthor Category	Jeroen Bax	0.968	0.345
	Mathew Farrer	1.000	0.125
	Filippo Marte	0.818	0.460
	Christodoulos Stefanadis	0.813	0.352
	*Macro Average*	0.900	0.321

**Figure 4:**
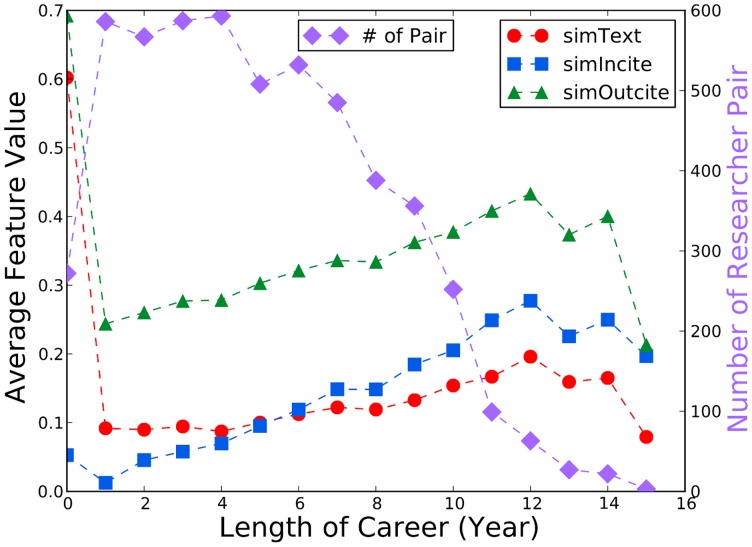
Research interest similarities over researcher career span. In the early stages of a researcher's career, collaborators with less research similarity are found but collaboration between two experienced researchers shows greater research interest similarity.

In our study we found that research interest features are an important feature category (Table 6). In contrast, such features were not studied extensively in other work. We therefore further analyzed their characteristics, such as their relation with author seniority. As shown in Figure 4, the research profile similarity features *simText*, *simIncite*, and *simOutcite* all increase as author seniority increases. Our results suggested that young researchers are more likely to collaborate with those whose research interests differ while senior or experienced researchers tended to collaborate with those whose research interests are close to them. In addition, the number of author pairs decreases as the authors get more senior, indicating fewer collaborations as the researchers become more senior. We did not show the data in which the authors have over 15 years of research because the data was very sparse.

## Discussion

### Models

The evaluation results for both the 10-fold cross-validation and the testing data (on the *RandomPairCategory* testing set) show that the logistic regression and SVM models are two best performing supervised machine learning models (logistic regression yielded an F1 score of 0.796 for 10-fold cross-validation and a score of 0.788 for testing while SVM produced 0.780 and 0.769 respectively, as shown in Tables 3 and 4). *IndividualAuthorCategory* is a more challenging evaluation data set as we tried to predict collaborators for individual researchers from the candidates that were collected from their close neighbors in the network. ARCR outperformed all the baselines with statistical significance (Table 5), which further shows that our model has the ability to recommend collaborators for the researcher. KNN model, which learns a non-linear decision boundary, did not perform as well as SVM and Logistic Regression as it only had an F1 score of 0.734 for the cross-validation on the training dataset and 0.726 on the *RandomPairCategory* testing set. This suggests that a linear decision boundary might be preferred.

In contrast, neither the naïve Bayes nor the naïve Bayes multinomial model performed well: an F1 score of 0.684 for 10-fold cross-validation and a score of 0.667 for the *RandomPairCategory* test set with the naïve Bayes model and an F1 score of 0.609 for 10-fold cross validation and 0.592 for the *RandomPairCategory* test set with the naïve Bayes multinomial model. The performance differences between logistic regression and the naïve Bayes and naïve Bayes multinomial models are both statistically significant (p<0.05, t-test). A possible reason for this under-performance is that both models assume conditional independence, which might not hold in our study. For example, *coauthorJaccard* and *numCommonCoauthor* are related, since they both depend on the number of common co-authors.

### Feature Analysis

Table 6 shows that the most important feature for collaboration prediction, according to information gain, is the similarity of out-citing citations, which represents an author's knowledge background. True positive instances tend to have a larger *simOutcite* value than negative instances (mean *simOutcite* is 0.305 for *RandomPairCategory* and 0.462 *IndividualAuthorCategory* positive instances while it is 0.159 and 0.264 for negative instances in the two categories respectively), suggesting that common background knowledge increases the chance for collaboration. As for the feature *simOutcite*, collaborating pairs have a higher *simText* score than non-collaborating pairs do. An author's publication history represents the author's research area and *simText* shows the similarity of two researchers' fields. Our results also show that research field overlap is positively related to potential collaborations.

MeSH terms can be considered the topics of a biomedical article, with the feature *simMeSH* a measure of research interest similarity. Therefore it is not surprising that *simMeSH* contributes to the classification. Keyword overlap was explored in [13] and was a top-ranking feature. In contrast to that study and [14] that did not explore text as features, we found that the feature *simMeSH* ranks below *simText* in information gain. We speculate that although MeSH terms represent an article's semantic content, they are not as robust as the bag of words formulation of *simText* for the task of author collaboration classification, because MeSH terms may not be considered as fine grained as word features in the abstract.

Our results also show that neighborhood structure plays an important role in predicting collaboration. The features *numCommonCoauthor*, *coauthorJaccard*, and *Adamic* all have large information gain. Note that [12] did not find *Adamic* is a useful feature. Positive instances tend to have larger number of common co-authors than negative instances, as for the *IndividualAuthorCategory* testing dataset, where researchers are topologically close (mean *numCommonCoauthor* is 1.0) but negative pairs still tend not to have common collaborators (the mean is closed to 0). Our results suggest that the strength of social ties is important for establishing collaboration. This conclusion is consistent with our hypothesis and previous findings, which show that common neighbors are a very effective predictor in social networks [12], [13].

Features that are related to researcher activity level, such as *sumCoauthor*, *sumRecency*, and *sumPub*, are ranked lower than *simOutcite*, *simText*, *coauthorJaccard*, *Adamic*, *numCommonCoauthor*, *simMeSH*, and *simIncite*, as measured by information gain, suggesting that two researchers' specific activities do not have to be closely related to establish a new collaboration. In contrast, the sum of co-authors was found to be among top features in [13], but it is not clear if this was influenced by the normalization by year, as carried out in our study. Consistent with previous findings, the clustering coefficient, which describes the transitivity of a collaboration, is not an effective feature [13]. It is also interesting to note that difference in seniority between collaborators, described by *diffSeniority*, has no impact on establishing a new collaboration in our approach.

**Figure 5:**
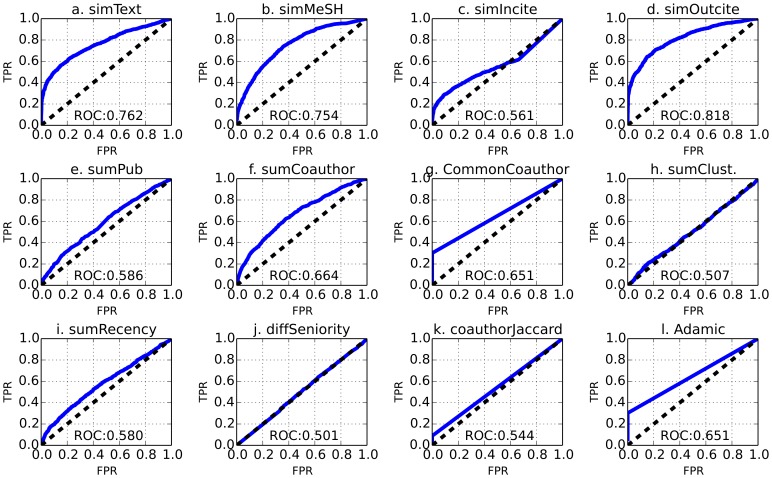
ROC for logistic regression classifiers trained by single feature. 1) Research interest features *simText*, *simMesh* and *simOutcite* (panels a, b and d) have large ROC areas, showing that they are informative for the classification. *simIncite* (panel c) however is not as large as other features in this category with 0.56 ROC only. 2) The ROC curves for common co-author based features (panels f, k and l) are a straight lines due to the fact that most of the author pairs (especially negative instances) don't have any common coauthors, and only 1/3 of the positive instances have non-zero common co-authors. 3) Other features such as *sumCoauthor* (panel f) is also effective for classification with 0.67 ROC. Activity feature *sumRecency* (panel i) has 0.60 ROC, and so does *sumPub* (panel e). *sumClusteringCoef* and *diffSeniority* (panels h, j) show only 0.53 and 0.54 ROC respectively. The individual ROC is consistent with information gain analysis, which also shows that the research interest features are most informative, followed by common neighbor based features.

Furthermore, we trained classifiers using every single feature individually and analyzed the performance as shown in Figure 5. 1) Research interest features *simText*, *simMesh* and *simOutcite* (Figure 5 panels *a*, *b* and *d*) have large ROC areas, showing that they are informative for the classification. *simIncite* (Figure 5*c*) however is not as large as other features in this category with 0.56 ROC only. 2) Common co-author based features (Figure 5 panels *f*, *k* and *l*) exhibit distinct patterns and are essentially equivalent features as they have large correlation coefficients among each other. For example *Adamic* and *numCommonCoauthor* have a correlation coefficient of 0.96. This suggests that we can use *numCommonCoauthor* as a feature and remove *Adamic*. The ROC curve for them is a straight line due to the fact that most of the author pairs (especially negative instances) don't have any common coauthors, and only 1/3 of the positive instances have non-zero common co-authors. 3) Other features such as *sumCoauthor* (Figure 5*f*) is also effective for classification with 0.67 ROC. Activity feature *sumRecency* (Figure 5*i*) has 0.60 ROC, and so does *sumPub* (Figure 5*e*). *sumClusteringCoef* and *diffSeniority* (Figure 5 panels *h*, *j*) show only 0.53 and 0.54 ROC respectively The individual ROC is consistent with information gain analysis, which also shows that the research interest features are most informative, followed by common neighbor based features.

There are inconsistency between the single feature logistic regression and information gain, and it is due to the fact that these two ranking mechanisms address feature contribution from slightly different perspectives. Information gain is the entropy difference of before and after splitting the data set by a specific value of this particular feature, and the entropy itself measures the level of impurity of the dataset. Single feature logistic regression, on the other hand, is essentially fitting the data by the particular feature. *simMeSH and sumCoauthor* is ranked lower than connectivity features (*numCommonCoauthor, coauthorJaccard* and *Adamic*) by information gain but higher than them by single feature logistic regression. The reason is that the above connectivity features have skewed distribution (almost all the negative instances don't have any common coauthors, and only 1/3 of the positive instances have non-zero common co-authors). Therefore it is easier to split the data set into two by value zero to yield high information gain. On the other hand *simMeSH* and *sumCoauthor* better fit the overall data due to their less skewed distributions.

In summary, previous work in author collaboration prediction mainly explored topological features. Our results, in contrast, show that in addition to topological features, semantic features are important. For example, we found that research interest is important for establishing a new collaboration. Specifically, research profile similarity features such as *simOutcite* and *simText*, as shown in Table 6, are the most important features–surpassing any of the topological features–for the classification. Tables 4 and 5 show that the supervised machine learning models that incorporate research similarity features significantly outperformed the baseline systems, which were built upon widely used topological features (*PreferentialAttachment*, *JaccardBaseline*). Possible interpretation is that knowing the other's work is a form of shared experience and the foundation of trust between two researchers. Their common knowledge, represented by the research similarity features, plays an important role for building collaboration.

As discussed earlier, although seniority plays a limited role in a collaboration, Figure 4 shows that when in their early career stage, researchers are more likely to collaborate with those whose research interests differ from theirs, suggesting that junior faculty are more open to collaborations. In contrast, collaborations between two senior researchers exhibit a higher degree of research interest similarity, suggesting that established researchers are more comfortable in their own fields and are less likely to initiate collaborations.

### Error Analysis

**Figure 6:**
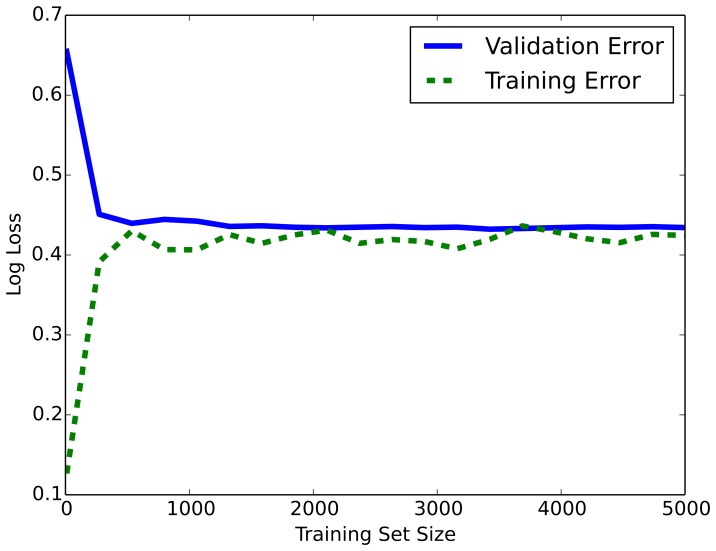
Learning curve for logistic regression with log loss metric. The training error and validation error converges by the time the dataset reaches the size of 1000 author pairs. Therefore the training set size (5361 positive instances and 5361 negative instances) is sufficient for the task.

In order to determine if our data size or features are sufficient, we analyzed the learning curve for the logistic regression model. The training set was split into two sets: training (66% of total instances) and validation set, and log loss is used for the error metric for the curve. As shown in Figure 6 the training error and validation error converges by the time the dataset reaches the size of 1000 author pairs. Therefore the training set size (5361 positive instances and 5361 negative instances) is sufficient for the task.

We also manually analyzed the prediction errors. We found that authors' publication history is important as many of our semantic features are derived from authors' research profile. If an author has few publications and few co-authors in the past, there is little information we can derive for features such as research interest, network topology, and productivity (or activity) level and therefore will not be able to predict accurately his/her future collaborators.

We found that the data incompleteness is one of the most important reasons for false negatives. The network that we used in this study is a sub-graph of MEDLINE publications only and therefore provides an incomplete picture of the publication history of certain authors. For example, Flaumenhaft R (author of PMID 12837380) has only one publication prior to 2009 with only one co-author. His/her pairing with Laurence RG (author of PMID 18715793) has a *simText* value of 0.010 and a *simOutcite* value of 0.143 (the average value for each feature in the positive training data was 0.134 and 0.325, respectively), although Laurence RG is more prolific in our network with 12 publications and 35 co-authors. In fact, by searching the larger database the MEDLINE we found that Flaumenhaft R has been publishing almost every year from 2003 to present and has many common co-authors with Laurence RG; this information was missing entirely in our network, which was built using the joint MEDLINE and Elsevier data only. As a result, our models predicted Flaumenhaft R was unlikely to collaborate with Laurence RG, which is therefore a false negative. On the other hand, false positive errors can arise due to the fact that these author pairs have features very much like those in the positive training data. These authors, however, might never have had a chance to actually know each other, leading to a false positive.

We also found that the noises in author name disambiguation contribute errors for both false positives and false negatives. Data sparseness arises when one author is mapped to two unique IDs by the author name disambiguation database we used. For example Guida M (author of PMID 17113552) has two IDs. There are only five publications assigned to the ID that we happened to use in our network, while there are 94 publications under the other ID. We are also aware that it is possible for an author to share the same ID with another, unrelated author; this can also cause a disambiguation error and the information from the unrelated author will be wrongly attributed to the original author. However, we did not actually find any such cases in our test sets.

Recall we have built two different testing data sets, and our analyses of true positive, false positive, true negative and false negative of the three testing data sets show interesting results (Table 7). In the *RandomPairCategory* dataset (i.e, positive and negative author pair data were randomly selected) our classification has low sensitivity (0.718) and high specificity (0.859) while *IndividualAuthorCategory* yielded the opposite (0.900 sensitivity and 0.321 specificity). The high specificity of *RandomPairCategory* is due to the fact that the negative instances are “very negative” as they were constructed by the random combination of two authors; therefore, they tend to share few research interests and even fewer common friends. In contrast, the negative instances of *IndividualAuthorCategory* testing set were from the sub-graph of the author, so they do have similar research interests and have a higher chance of sharing a common collaborator. The sensitivity advantage of *IndividualAuthorCategory* can be understood in a similar way, as the positive instances, which were sampled from the sub-graph of the author, are “very positive” and share research interests and common collaborators, which increases the likelihood of the classifiers to classify them as positive.

### Limitations

There are several limitations to this study. First, we did not explore learning features of broad social factors, including institutional policies like the status of an IRB application or institution-specific restrictions, because it is difficult to obtain these data. Second, our data are incomplete and contain missing information. We used a sub-graph of the MEDLINE co-author network and therefore the author publication histories may not be complete, as we described in the error analysis. Missing publications indicates missing the important research interest information. It also takes time for an article to accumulate citations, so *simIncite* may be biased to have more citations for older works than for recent ones. Finally, our training and testing period time cutoff is ad-hoc, and we define a negative instance pair as authors who did not collaborate by the time of the training or testing period, which might not be true in reality for every pairs.

### Future Work

We identify the following directions for future research. First, we would like to incorporate all the available MEDLINE records to minimize the challenges of missing data. Secondly, we would like to explore additional learning features including the funding status, the collaboration strength, and the impact of an article. Thirdly, it is important to analyze in depth the research collaboration network and its topological characteristics. For example, interdisciplinary collaboration may involve a sub-graph (inter-group collaboration) that may exhibit different characteristics from the overall graph. Finally we may explore other machine learning models, including collaborative filtering.

## Conclusions

In this study we applied and evaluated four established supervised machine-learning models, namely naïve Bayes, naïve Bayes multinomial, SVMs, and logistic regression, and explored rich learning features for automatic research collaboration prediction. We found supervised machine learning models can predict research collaboration with a high performance with an ROC ranging from 0.766 to 0.980 on different datasets, and logistic regression and SVMs performed the best. In addition, we identified three key factors for establishing new collaboration: research interest, common collaborators, and research productivity. Our research is important as it not only produces an important tool for automatic author collaboration prediction, but also contributes significantly to the science of evolving network modeling.
